# How does social media addiction make you fear exercise? The relationship between social media addiction and kinesiophobia among college students: the mediating role of social physique anxiety

**DOI:** 10.3389/fpsyg.2026.1840700

**Published:** 2026-05-20

**Authors:** Ying Fang Zhang, Tao Chen

**Affiliations:** Xinjiang University of Technology, Hotan, Xinjiang Uyghur Autonomous Region, China

**Keywords:** colledge student, kinesiophobia, mediating effect, social media addiction, social physique anxiety

## Abstract

**Objective:**

As social media becomes deeply embedded in daily life, concerns have emerged regarding its influence on health-related behaviors. The present study examines how social media addiction relates to kinesiophobia among Chinese college students, with particular attention to the mediating role of social physique anxiety.

**Methods:**

A cross-sectional study design was employed, involving 395 college students (age range 18–21 years, all first-year students). Measurements were conducted using the Bergen Social Media Addiction Scale (BSMAS), the Social Physique Anxiety Scale (SPAS-15), and the Tampa Scale for Kinesiophobia (TSK). Body mass index (BMI) was calculated based on self-reported height and weight. Pearson correlation analysis was used to examine relationships among the variables, and mediation analysis was performed using the PROCESS macro (Model 4) for SPSS, with gender, age, and BMI included as control variables. The significance of the mediation effect was tested using 5,000 bootstrap samples.

**Results:**

(1) Social media addiction was significantly positively correlated with kinesiophobia (*r* = 0.229, *p* < 0.001). (2) Social media addiction was significantly positively correlated with social physique anxiety (*r* = 0.370, *p* < 0.001), and social physique anxiety was significantly positively correlated with kinesiophobia (*r* = 0.299, *p* < 0.001). (3) Mediation analysis revealed that social physique anxiety played a significant partial mediating role in the relationship between social media addiction and kinesiophobia [indirect effect = 0.045, 95% CI (0.022, 0.071)], with the mediation effect accounting for 40.41% of the total effect.

**Conclusion:**

The findings suggest that social media addiction is associated with exercise avoidance both directly and indirectly through heightened concerns about body-related evaluation. Due to the cross-sectional design, causal inferences cannot be drawn. These results highlight the role of psychosocial factors in shaping exercise-related fear.

## 1 Introduction

Kinesiophobia has traditionally been defined as an excessive and debilitating fear of physical movement, in which activity is perceived as a potential source of pain or reinjury (Kori et al., 1990; Miller et al., 1991). Previous research has primarily focused on clinical populations, particularly individuals with chronic pain or injury, such as patients undergoing rehabilitation (Liu et al., 2024; Lucidi et al., 2025) and athletes recovering from injury (Park et al., 2024). In these contexts, kinesiophobia has also been identified as an important predictor of return to sport (Lucidi et al., 2025).

From a theoretical perspective, the fear-avoidance model has been widely recognized as a key mechanism underlying kinesiophobia (Bullock et al., 2022). A substantial body of research indicates that chronic pain is closely associated with mental health problems and often co-occurs with psychological distress, sharing common neural mechanisms and intervention pathways (Kohrt et al., 2018; Hooten, 2016; Goesling et al., 2018). These conditions may further affect individuals’ behavioral patterns and social functioning (Corser et al., 2023; Klem et al., 2025). Building on this evidence, it is plausible that kinesiophobia toward physical activity may not be exclusively driven by physical pain, but could also arise from psychological or social factors. Importantly, in non-clinical college students, elevated TSK scores may reflect exercise-related fear or avoidance tendencies rather than clinically defined kinesiophobia rooted in pain or injury.

In the context of rapid digitalization, social media has become deeply embedded in the daily lives of young adults. Global data indicate that nearly 5 billion people were active social media users by 2025, with average daily usage exceeding 2 h (Statista, 2026). While moderate use may facilitate communication and access to health information, excessive use has been associated with a range of negative psychological outcomes, including anxiety, depression, and body image concerns (Conte et al., 2025; Wang et al., 2025; Huang et al., 2025). A growing body of research suggests that frequent exposure to idealized body images on social media platforms can lead to upward social comparison, internalization of unrealistic appearance standards, and body dissatisfaction (Karam et al., 2023; Çakmak and Tanrıöver, 2024).

Furthermore, problematic social media use has been found to be negatively associated with physical activity levels (Qin et al., 2025; Siddik et al., 2025). One possible explanation is that excessive social media use heightens individuals’ sensitivity to appearance-related evaluation (Brown and Tiggemann, 2016; Prichard et al., 2020). In environments such as gyms or sports fields, where body exposure and social comparison are salient, individuals may perceive these settings as socially evaluative or even threatening (Blond, 2008; Grabe et al., 2008). From a fear-avoidance perspective, such perceived threats may lead to avoidance tendencies toward physical activity.

Social physique anxiety (SPA) provides a useful framework for understanding this process. It refers to the anxiety individuals experience when they believe their physique is being evaluated by others, particularly in situations involving body exposure (Hart et al., 1989). Empirical studies have shown that SPA is negatively associated with physical activity participation (Herring et al., 2021; Portman et al., 2018; Jiang and Wang, 2025). Importantly, SPA is distinct from general social anxiety. While social anxiety reflects a broad fear of negative evaluation in social interactions, SPA specifically concerns body-related evaluation. This distinction is particularly relevant in physical activity contexts, where body exposure and appearance-based comparison are salient. Therefore, SPA may represent a more proximal psychological factor influencing exercise-related behaviors (Pritchard et al., 2021).

From a theoretical standpoint, self-discrepancy theory suggests that discrepancies between one’s actual self and ideal self may evoke negative emotional states such as anxiety and dissatisfaction (Higgins et al., 1986). Social media environments, characterized by frequent exposure to idealized body images, may amplify these discrepancies. In addition, self-objectification theory suggests that individuals in appearance-focused environments are more likely to adopt an observer’s perspective toward their bodies, thereby increasing body-related anxiety (Sarda et al., 2025; Çimke and Yıldırım, 2023). Moreover, social pain theory posits that the psychological distress induced by social evaluation or exclusion shares similar neural mechanisms with physical pain (Kapos et al., 2024). Thus, anticipating negative evaluation in exercise contexts may lead to psychological discomfort and reduced engagement in physical activity.

Based on the above, the present study does not attempt to redefine kinesiophobia, but rather explores whether fear-avoidance mechanisms may also operate in psychosocial contexts. Specifically, this study examines the association between social media addiction and kinesiophobia among college students and further investigates the mediating role of social physique anxiety. By integrating perspectives from fear-avoidance theory and body image research, this study aims to provide a more focused understanding of exercise avoidance behaviors in non-clinical populations.

**Figure 1:**
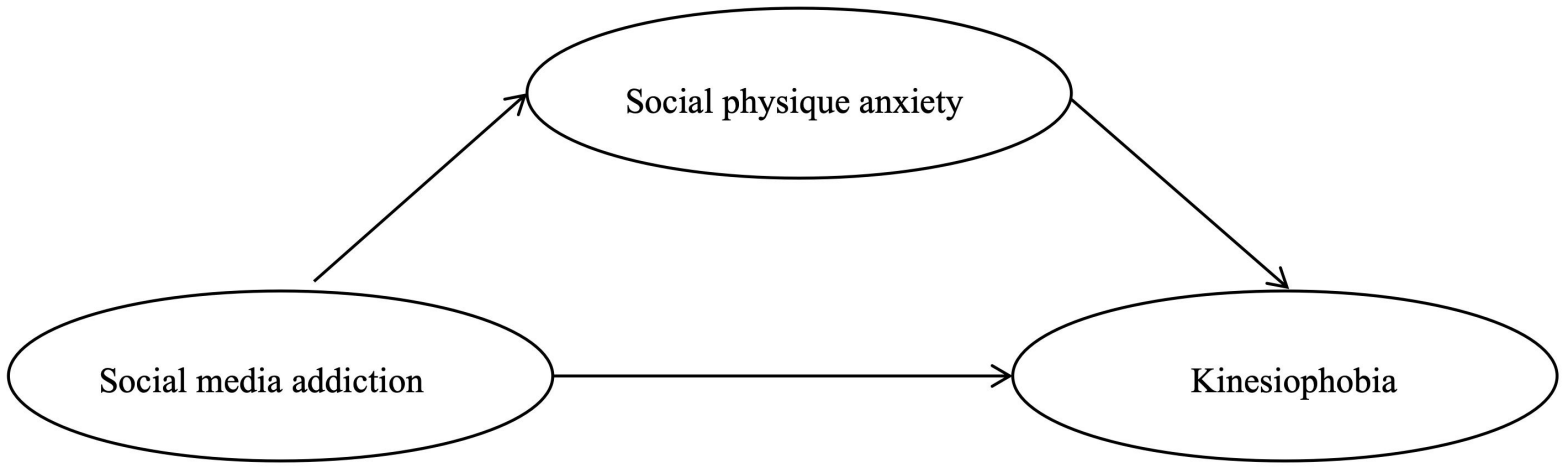
The hypothesized model.

Accordingly, the following hypotheses are proposed and constructing a mediation model (Figure 1):

*H1*: Social media addiction is positively associated with kinesiophobia.

*H2*: Social physique anxiety is positively associated with kinesiophobia.

*H3*: Social physique anxiety mediates the relationship between social media addiction and kinesiophobia.

## 2 Methods

### 2.1 Participants

Prior to data collection, an *a priori* power analysis was conducted using G*Power 3.1 (Faul et al., 2009) to determine the minimum required sample size. Given that the primary analyses involved correlation and regression-based mediation models, the “Linear multiple regression: Fixed model, *R*^2^ deviation from zero” procedure was selected. Based on Cohen’s (1988) guidelines, a medium effect size (*f*^2^ = 0.15), a significance level of *α* = 0.05, a statistical power of 0.80, and four predictors were specified. The analysis indicated a minimum required sample size of 85 participants. The final sample size of 395 therefore exceeded this requirement, suggesting adequate statistical power.

A cross-sectional design was employed. Participants were recruited using convenience sampling from a university of science and engineering. The sample consisted of first-year college students aged between 17 and 21 years (*M* = 18.71, SD = 0.88). A total of 425 questionnaires were distributed, and 395 valid responses were retained (valid response rate = 92.94%). The final sample included 323 males (81.8%) and 72 females (18.2%).

Prior to data collection, participants attended an offline briefing session during which the purpose, procedures, and ethical considerations of the study were explained. Written informed consent was obtained from all participants. Questionnaires were completed anonymously via an online survey platform in a controlled classroom setting to reduce external distractions. No personally identifiable information was collected.

This study was conducted in accordance with the Declaration of Helsinki and was approved by the institutional ethics committee.

### 2.2 Measures

#### 2.2.1 Social media addiction (SMA)

Social media addiction was measured using the Bergen Social Media Addiction Scale (BSMAS), developed by Andreassen et al. (2017). The scale was used to assess participants’ social media use and the degree of addictive behavior over the past year. The BSMAS consists of 18 items, with sample items such as “Used social media to reduce feelings of restlessness.” Each item is rated on a 5-point Likert scale ranging from 1 (never) to 5 (very often). The scale has been widely used in the academic literature, and previous research has demonstrated satisfactory reliability and validity among adolescents in mainland China (Hou et al., 2019). In this study, the Cronbach’s *α* coefficient for the scale was 0.927.

#### 2.2.2 Social physique anxiety (SPA)

Social physique anxiety was assessed using the Social Physique Anxiety Scale (SPAS-15), which was adapted for the Chinese population (Xu, 2003). The scale comprises three dimensions: worry about negative evaluation from others (6 items), discomfort with physical self-presentation (6 items), and anxiety about social comparison (3 items). Items are rated on a 5-point Likert scale, with higher scores indicating greater levels of social physique anxiety. The scale has been made publicly available by the author for free use. In this study, the Cronbach’s *α* coefficient for the scale was 0.846, indicating good reliability.

#### 2.2.3 Tampa Scale for Kinesiophobia (TSK)

The Tampa Scale for Kinesiophobia (TSK) was originally developed by Miller et al. (1991) for use in the field of pain research to assess patients’ level of fear of movement in response to pain-related stimuli. The Chinese version of the scale was translated and validated by Hu (2012), with a Cronbach‘s α coefficient of 0.778, indicating satisfactory reliability. The Chinese version of the TSK consists of 17 items rated on a 4-point Likert scale, with items 4, 8, 12, and 16 reverse-scored. Total scores range from 17 to 68, with higher scores indicating a greater degree of kinesiophobia and a higher likelihood of exercise avoidance behavior. Although the TSK was originally developed to assess fear of movement related to pain and injury, in this non-clinical sample we interpret higher TSK scores as indicating a general tendency to avoid physical activity, without implying that this avoidance is necessarily pain-driven. The concurrent measurement of social physique anxiety allows us to empirically separate the variance attributable to evaluative concerns.”

### 2.3 Control variables

Gender, age, and body mass index (BMI) were included as control variables. BMI was calculated based on self-reported height and weight using the standard formula (kg/m^2^). Participants were categorized into underweight, normal weight, overweight, and obese groups according to established classification criteria.

### 2.4 Statistical analysis

A total of 395 valid samples were included in the final analysis. Data analysis was performed using SPSS 29.0. Descriptive statistics were first computed for all demographic variables. Pearson correlation analysis was then conducted to examine the relationships among demographic variables, social media addiction, social physique anxiety, and kinesiophobia. To test the hypothesized mediation model in which social physique anxiety mediates the relationship between social media addiction and kinesiophobia, Model 4 of the PROCESS macro for SPSS (version 4.1) was applied (Hayes, 2018). Model 4 was selected because it is specifically designed for single-mediator models, allowing simultaneous estimation of direct, indirect, and total effects, and provides robust statistical inference via bias-corrected bootstrapping, which is the most recommended method for examining mediation effects. The significance of the indirect effect was examined using a bias-corrected bootstrapping procedure with 5,000 resamples. An indirect effect was considered statistically significant if its 95% corrected confidence interval (CI) did not include zero (Hayes, 2018).

## 3 Result

### 3.1 Common method bias test

Given that all variables in this study were measured via participants’ self-reports on the self-report questionnaires, there was a potential risk of common method bias affecting the results. To assess this possibility, Harman’s single-factor test was conducted on all measurement items using an unrotated exploratory factor analysis. The analysis extracted nine factors with eigenvalues greater than 1, with the first factor accounting for 25.03% of the total variance—below the commonly accepted threshold of 40%. This result suggests that common method bias is unlikely to fully account for the observed relationships. However, given the limited sensitivity of Harman’s single-factor test, common method bias cannot be completely ruled out. Therefore, the findings should be interpreted with caution.

### 3.2 Descriptive statistics

A total of 395 valid questionnaires were included in the final analysis. Participants were first-year college students aged between 17 and 21 years (*M* = 18.71, SD = 0.88). The sample consisted of 323 males (81.8%) and 72 females (18.2%). In terms of body mass index (BMI), 57.7% of participants were classified as normal weight, while 14.4% were underweight, 20.0% were overweight, and 7.6% were obese. For the main study variables, the mean score of social media addiction was 38.03 (SD = 10.47), the mean score of social physique anxiety was 44.56 (SD = 8.77), and the mean score of kinesiophobia was 39.75 (SD = 5.18). Based on the cutoff score (≥ 38), 71.9% of participants were classified as having high levels of kinesiophobia (see Table 1).

**Table 1:** Descriptive statistics.

Variable	Category	*n*	MEAN	SD
Gender	Male	323 (81.8%)		
	Female	72 (18.2%)		
AGE	<18	3 (0.8%)	18.71	0.88
	18–20	384 (98.9%)		
	>20	8 (0.3%)		
BMI	Underweight (<18.5)	57 (14.4%)	22.2	3.69
	Normal weight (18.5–23.9)	228 (57.7%)		
	Overweight (24.0–27.9)	79 (20%)		
	Obesity (≥28.0)	30 (7.6%)		
SMA		395	38.03	10.47
SPA		395	44.56	8.769
TSK		395	39.75	5.182
	Low (17–37)	111 (28.1%)		
	High (38–68)	284 (71.9%)		

### 3.3 Correlation coefficients

Pearson correlation analysis was conducted to examine the relationships among the study variables (see Table 2). Social media addiction was significantly positively correlated with kinesiophobia (*r* = 0.229, *p* < 0 0.001) and social physique anxiety (*r* = 0.370, *p* < 0 0.001). Social physique anxiety was also significantly positively correlated with kinesiophobia (*r* = 0.299, *p* < 0.001). In addition, gender was significantly correlated with social physique anxiety (*r* = 0.239, *p* < 0.001). No significant correlations were found between age or BMI and the main study variables.

**Table 2:** Correlation coefficients.

	SMA	TSK	SPA	AGE	Gender	BMI
SMA	1					
TSK	0.229**	1				
SPA	0.370**	0.299**	1			
AGE	−0.031	−0.047	−0.013	1		
Gender	0.025	0.057	0.239**	−0.076	1	
BMI	−0.059	−0.042	−0.075	−0.018	−0.227**	1

**p < 0.001.

### 3.4 Mediating role of social physique anxiety

**Figure 2:**
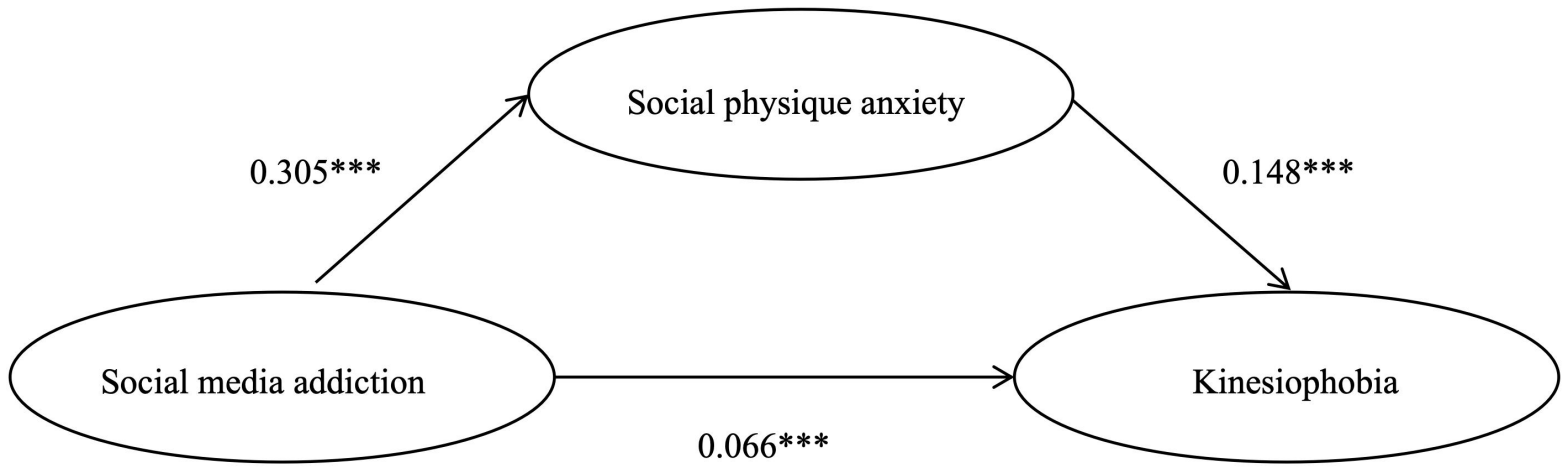
Model of social physique anxiety as a mediating role between social media addiction and kinesiophobia. **p > <0.001. ***p < 0.001.

The mediating effect of social physique anxiety was tested using PROCESS macro Model 4, with gender, age, and BMI included as control variables (see Table 3). The results showed that social media addiction was significantly positively associated with social physique anxiety (*B* = 0.305, *p* < 0.001). When both social media addiction and social physique anxiety were entered simultaneously into the regression model predicting kinesiophobia, both variables remained statistically significant (social media addiction: *B* = 0.067, *p* < 0.01; social physique anxiety: *B* = 0.148, *p* < 0 0.001). None of the control variables showed significant associations with kinesiophobia. The indirect effect was examined using a bootstrap procedure with 5,000 resamples. The indirect effect of social media addiction on kinesiophobia through social physique anxiety was 0.045, with a 95% confidence interval of [0.022, 0.071], which did not include zero (see Table 4). The indirect effect accounted for 40.41% of the total effect,thereby supporting Hypothesis H3 (Figure 2).

**Table 3:** The mediating role of social physique anxiety.

Variables	*B*	SE	*t*	*p*	LLCI	ULCI	*R* ^2^
OUTCOME VARIABLE: SPA							0.1898
SMA	0.3053	0.0383	7.9773	0	0.23	0.3805	
AGE	0.1578	0.4545	0.3471	0.7287	0.7359	1.0514	
Gender	5.2386	1.0653	4.9177	0	3.1442	7.333	
BMI	−0.001	0.1114	−0.0094	0.9925	−0.22	0.218	
OUTCOME VARIABLE: TSK							0.1074
SMA	0.0665	0.0256	2.5942	0.0098	0.0161	0.1169	
SPA	0.1479	0.0314	4.7018	0	0.086	0.2097	
AGE	−0.2407	0.2823	−0.8525	0.3944	−0.7958	0.3144	
Gender	−0.1793	0.6818	−0.263	0.7927	−1.5197	1.1611	
BMI	−0.0267	0.0692	−0.3855	0.7001	−0.1627	0.1093	

**Table 4:** Total effect, direct effect and mediating effect.

Effect type	Effect	SE	LLCI	ULCI	% of total effect
Direct effect	0.0665	0.0256	0.0161	0.1169	59.59%
Indirect effect	0.0451	0.0126	0.0222	0.0712	40.41%
Total effect	0.1116	0.0244	0.0637	0.1596	100%

To further explore whether gender moderated the relationships among the study variables, an additional moderated mediation analysis was conducted using PROCESS Model 59, with age, and BMI included as control variables. The interaction terms involving gender were not statistically significant (*p* > 0.05), indicating that gender did not significantly moderate the association between social media addiction, social physique anxiety, and kinesiophobia in the present sample.

## 4 Discussion

Drawing on the fear-avoidance model and body image-related theories, the present study examined the relationships among social media addiction, social physique anxiety, and kinesiophobia in a sample of college students. The findings indicated that social media addiction was positively associated with kinesiophobia, and that social physique anxiety partially mediated this relationship. Importantly, given the cross-sectional nature of the data, these findings should be interpreted as associations rather than causal relationships.

First, the results revealed a significant positive association between social media addiction and kinesiophobia. This finding is consistent with previous research suggesting that excessive social media use is linked to a range of negative psychological and behavioral outcomes, including reduced physical activity (Qin et al., 2025; Siddik et al., 2025; Che et al., 2025) and increased emotional distress (Nguyen et al., 2025). One possible explanation is that prolonged exposure to idealized body images on social media may promote upward social comparison and internalization of unrealistic appearance standards (Karam et al., 2023; Çakmak and Tanrıöver, 2024). As a result, individuals may become more sensitive to body-related evaluation in real-world exercise contexts. In environments such as gyms or sports fields, where body exposure is salient, individuals may anticipate negative evaluation and perceive these situations as socially threatening (Blond, 2008; Grabe et al., 2008). From the perspective of the fear-avoidance model, when individuals interpret a situation as threatening, they may experience fear and subsequently engage in avoidance behaviors (Wideman et al., 2009). In this sense, the present findings suggest that fear-avoidance–like responses may also emerge in contexts involving social-evaluative concerns, rather than physical pain alone.

Second, the mediation analysis indicated that social physique anxiety played a significant role in linking social media addiction to kinesiophobia. This finding suggests that social media use may be associated with exercise avoidance indirectly through increased concerns about body evaluation. This mechanism can be understood through several theoretical perspectives. According to self-discrepancy theory, discrepancies between one’s actual and ideal self can elicit negative emotional states such as anxiety and dissatisfaction (Higgins et al., 1986; Mason et al., 2019). Social media environments, characterized by frequent exposure to idealized body images, may amplify such discrepancies, thereby increasing vulnerability to social physique anxiety (Söyünmez et al., 2024). In addition, self-objectification theory posits that individuals in appearance-focused environments are more likely to adopt an observer’s perspective toward their bodies, which may contribute to body dissatisfaction and body-related anxiety (Sarda et al., 2025; Saunders et al., 2024). Furthermore, social pain theory suggests that distress arising from social evaluation or exclusion may share similarities with physical pain (Kapos et al., 2024; MacDonald and Leary, 2005). Taken together, these perspectives support the interpretation that concerns about social evaluation may contribute to discomfort in exercise contexts, thereby increasing the likelihood of avoidance tendencies.

It is worth noting that although the mediating effect of social physique anxiety was statistically significant, the observed correlations among variables were relatively modest (e.g., *r* = 0.229–0.370). This indicates that the relationships, while meaningful, are not strong and that kinesiophobia is likely influenced by multiple factors beyond those examined in this study. In addition to body-related anxiety, other mechanisms—such as emotional states (e.g., anxiety and depression), behavioral displacement (e.g., reduced time for physical activity), and maladaptive cognitions—may also contribute to exercise avoidance (Jing et al., 2025; Purba et al., 2023; Fardouly et al., 2015). Future research is encouraged to incorporate these variables into more comprehensive models.

Furthermore, social media addiction retained a significant direct association with kinesiophobia after accounting for social physique anxiety, suggesting the presence of additional pathways. For instance, emotional distress associated with problematic social media use may reduce motivation for physical activity (Jing et al., 2025), while excessive screen time may displace opportunities for exercise and promote sedentary behavior (Purba et al., 2023). These findings highlight the complexity of exercise avoidance behaviors and underscore the need for multifactorial explanations.

Although gender differences are theoretically relevant in this context, particularly given prior evidence that females tend to report higher levels of social physique anxiety (Alcaraz-Ibáñez et al., 2023; Zartaloudi et al., 2023), the moderation effects of gender were not statistically significant in the present study. One likely explanation is the substantial gender imbalance in the sample, which was predominantly male. This imbalance may have reduced the statistical power to detect potential interaction effects. In addition, it is possible that gender differences in this context are more strongly reflected in mean-level differences rather than in moderating the relationships between variables. Therefore, the absence of significant moderation effects should be interpreted with caution. Future research should employ more balanced and representative samples and adopt more rigorous analytical approaches, such as multi-group analysis or moderated mediation models, to further explore whether the pathways linking social media addiction, social physique anxiety, and kinesiophobia differ by gender.

## 5 Theoretical contributions

From a theoretical perspective, this study contributes to the literature by integrating the fear-avoidance model with body image–related frameworks in a non-clinical population. Rather than redefining kinesiophobia, the present findings suggest that similar avoidance patterns may occur when perceived threats are related to social evaluation and body image concerns. In addition, by identifying social physique anxiety as a mediating variable, this study provides a more specific psychological pathway linking social media experiences to exercise-related behavior. This integration contributes to a more nuanced understanding of how digital environments may shape health-related behaviors.

## 6 Implications for practice

From a practical standpoint, the findings suggest that interventions aimed at promoting physical activity among college students should not only focus on increasing motivation, but also address psychological barriers related to body evaluation. For example, creating supportive and non-evaluative exercise environments may help reduce anxiety associated with body exposure. Additionally, promoting media literacy and encouraging critical engagement with social media content may help mitigate unrealistic body standards and reduce social comparison tendencies. Psychological interventions, such as self-compassion training or cognitive-behavioral strategies, may also be beneficial for individuals with high levels of social physique anxiety.

## 7 Limitations and future research

Several limitations should be acknowledged. First, the cross-sectional design precludes causal inference; therefore, future studies should employ longitudinal or experimental designs to clarify the temporal direction of the observed relationships. Second, the sample was predominantly male, which may limit the generalizability of the findings to other populations. Third, all variables were measured using self-report instruments, raising the potential for common method bias. Although Harman’s single-factor test suggested that common method bias was not a major concern, this test has limited sensitivity, and future research should adopt more robust methodological approaches. Finally, while gender differences were observed in social physique anxiety, the moderating role of gender was not formally tested and warrants further investigation. In addition, the substantial gender imbalance in the sample may have constrained the ability to detect potential moderation effects, particularly those involving gender differences in the relationships among the key variables.

## 8 Conclusion

This study examined the relationships among social media addiction, social physique anxiety, and kinesiophobia in college students. The results indicated that social media addiction was positively associated with kinesiophobia, and that social physique anxiety partially mediated this relationship, accounting for 40.41% of the total effect.

These findings suggest that exercise avoidance tendencies may be associated with both direct effects of social media use and indirect effects through body-related evaluative concerns. Importantly, rather than redefining kinesiophobia, the results indicate that fear-avoidance–like responses may also be observed in contexts involving social evaluation and body image concerns.

From a theoretical perspective, the study identifies a potential psychological pathway linking social media experiences to exercise-related behavior. From a practical perspective, the findings highlight the importance of addressing body-related anxiety and social evaluative concerns in efforts to promote physical activity among college students.

Given the cross-sectional design and the relatively modest effect sizes, the findings should be interpreted with caution. Future research is needed to further examine these relationships using longitudinal or experimental approaches.

## Data Availability

The raw data supporting the conclusions of this article will be made available by the authors, without undue reservation.
